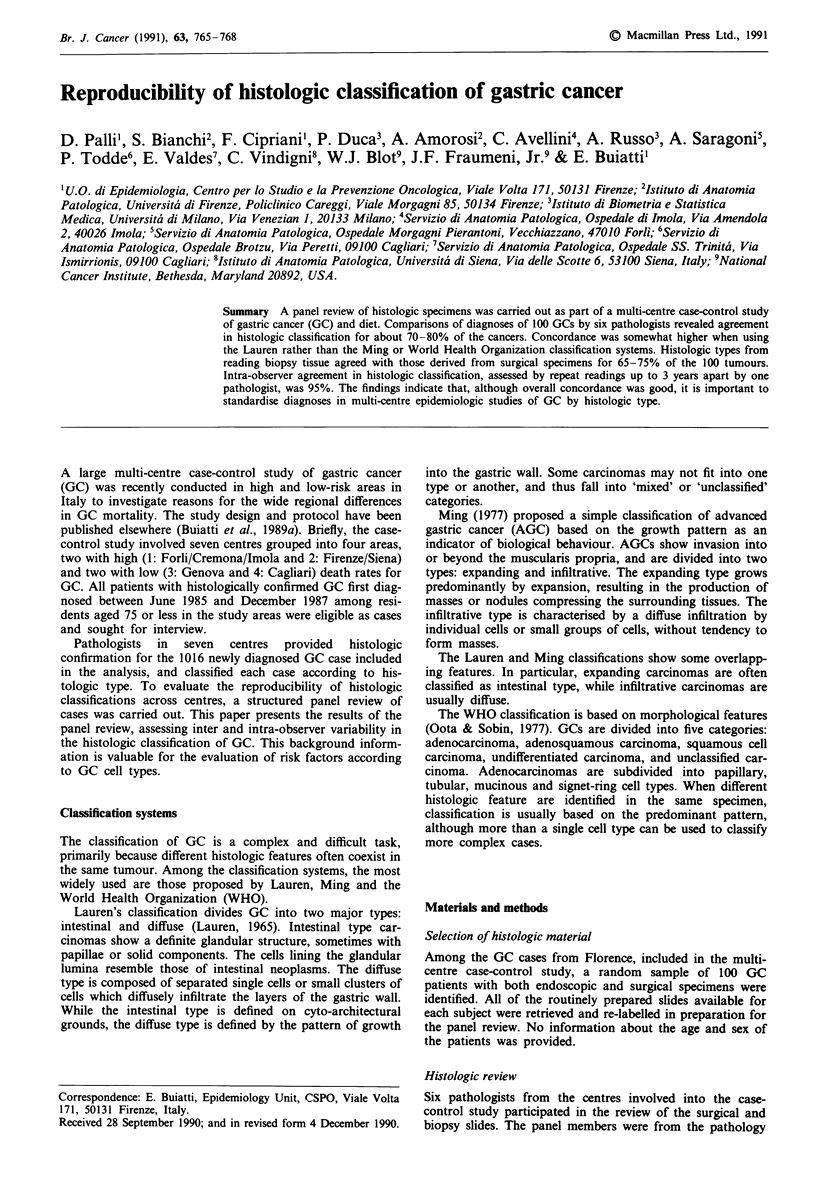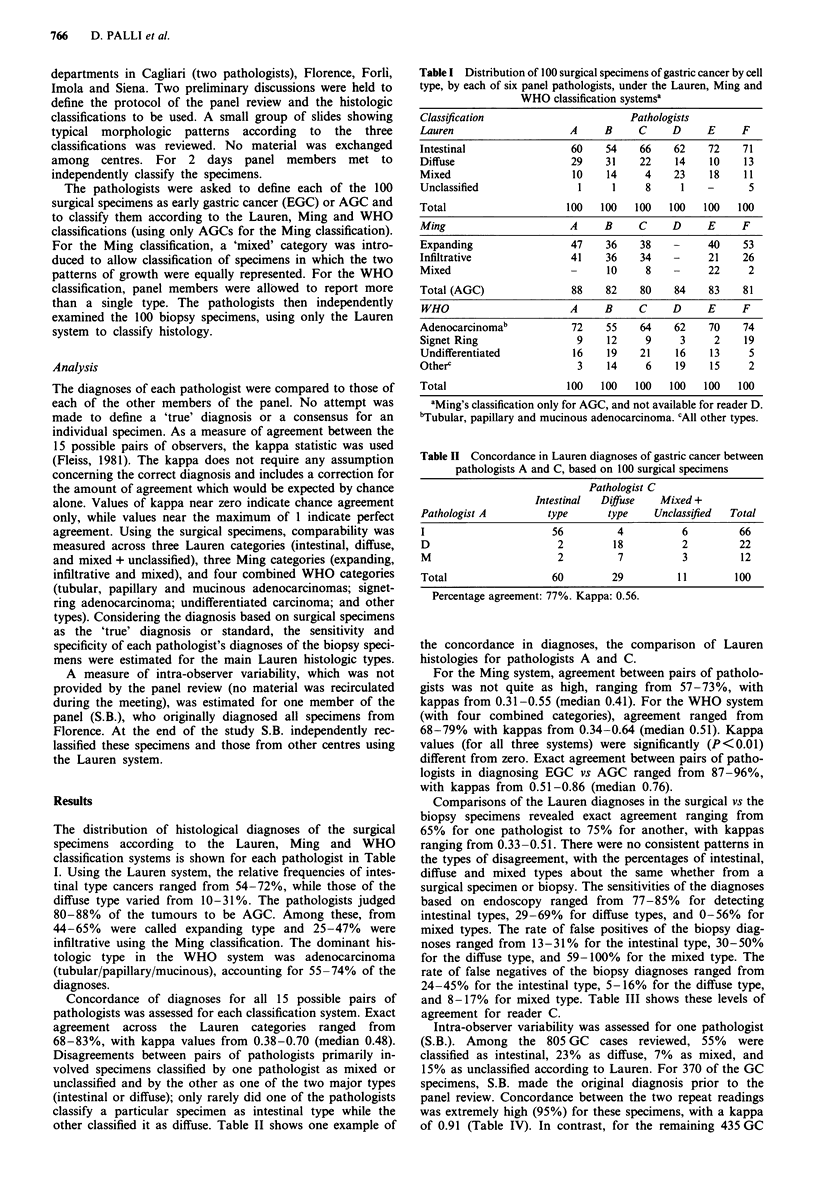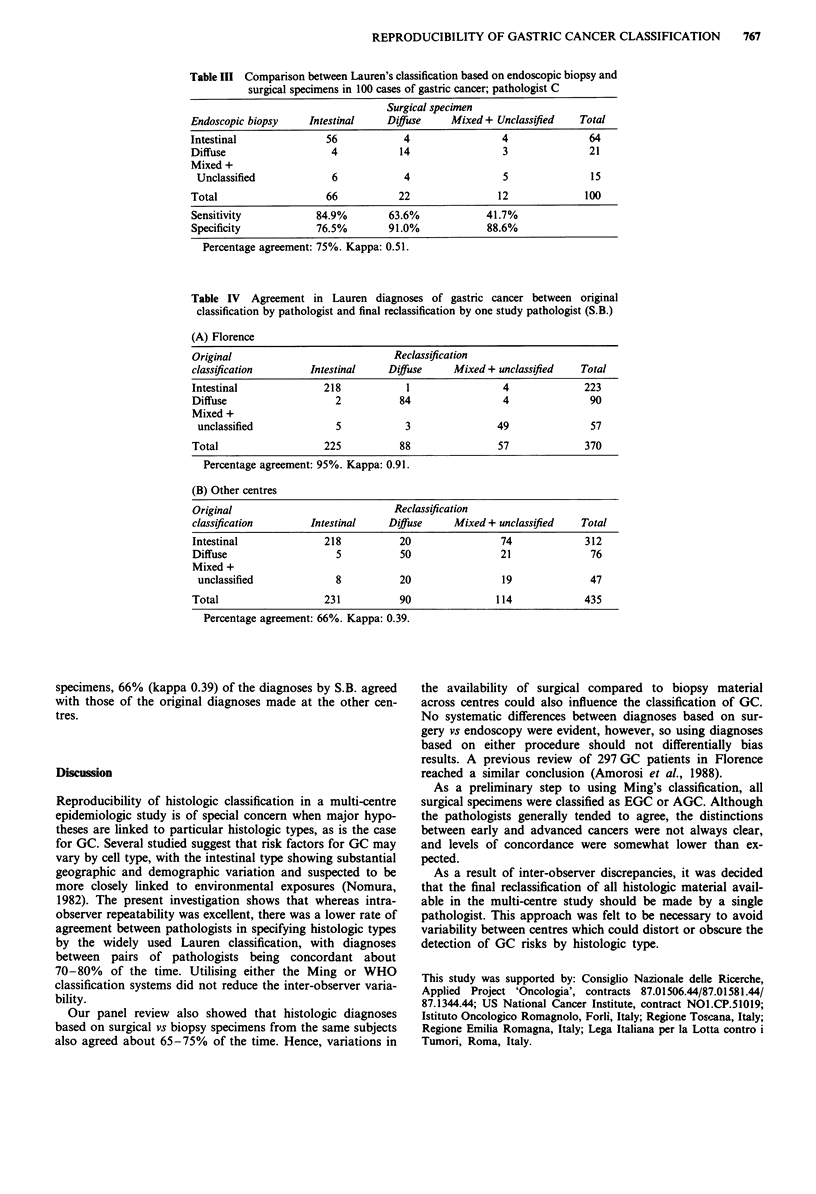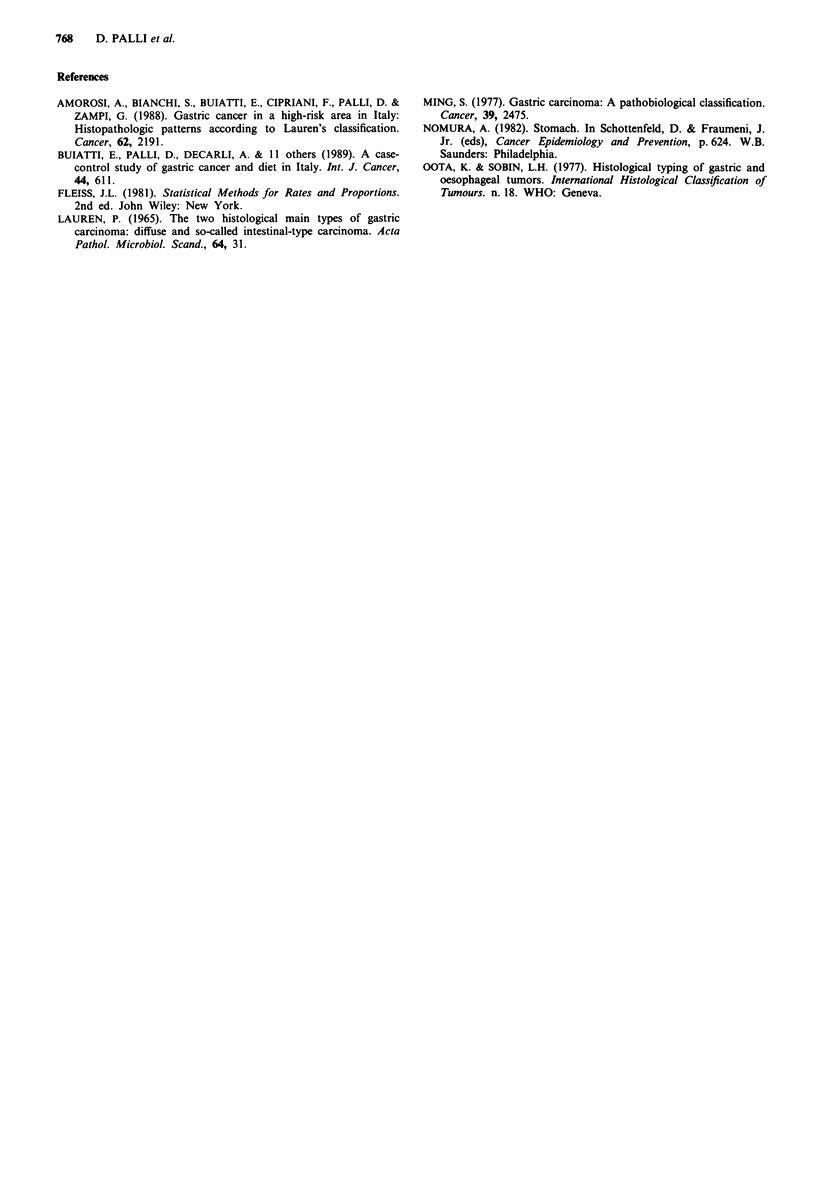# Reproducibility of histologic classification of gastric cancer.

**DOI:** 10.1038/bjc.1991.171

**Published:** 1991-05

**Authors:** D. Palli, S. Bianchi, F. Cipriani, P. Duca, A. Amorosi, C. Avellini, A. Russo, A. Saragoni, P. Todde, E. Valdes

**Affiliations:** U.O. di Epidemiologia, Centro per lo Studio e la Prevenzione Oncologica, Firenze, Italy.

## Abstract

A panel review of histologic specimens was carried out as part of a multi-centre case-control study of gastric cancer (GC) and diet. Comparisons of diagnoses of 100 GCs by six pathologists revealed agreement in histologic classification for about 70-80% of the cancers. Concordance was somewhat higher when using the Lauren rather than the Ming or World Health Organization classification systems. Histologic types from reading biopsy tissue agreed with those derived from surgical specimens for 65-75% of the 100 tumours. Intra-observer agreement in histologic classification, assessed by repeat readings up to 3 years apart by one pathologist, was 95%. The findings indicate that, although overall concordance was good, it is important to standardise diagnoses in multi-centre epidemiologic studies of GC by histologic type.


*Br. J. Cancer* (1991), **63**, 765–768

© Macmillan Press Ltd., 1991

### Reproducibility of histologic classification of gastric cancer

D. Palli[1], S. Bianchi[2], F. Cipriani[1], P. Duca[3], A. Amorosi[2], C. Avellini[4], A. Russo[3], A. Saragoni[5], P. Todde[6], E. Valdes[7], C. Vindigni[8], W.J. Blot[9], J.F. Fraumeni, Jr.[9] & E. Buiatti[1]

[1]*U.O. di Epidemiologia, Centro per lo Studio e la Prevenzione Oncologica, Viale Volta 171, 50131 Firenze;* [2]*Istituto di Anatomia Patologica, Universitá di Firenze, Policlinico Careggi, Viale Morgagni 85, 50134 Firenze;* [3]*Istituto di Biometria e Statistica Medica, Universitá di Milano, Via Venezian 1, 20133 Milano;* [4]*Servizio di Anatomia Patologica, Ospedale di Imola, Via Amendola 2, 40026 Imola;* [5]*Servizio di Anatomia Patologica, Ospedale Morgagni Pierantoni, Vecchiazzano, 47010 Forlì;* [6]*Servizio di Anatomia Patologica, Ospedale Brotzu, Via Peretti, 09100 Cagliari;* [7]*Servizio di Anatomia Patologica, Ospedale SS. Trinitá, Via Ismirrionis, 09100 Cagliari;* [8]*Istituto di Anatomia Patologica, Universitá di Siena, Via delle Scotte 6, 53100 Siena, Italy;* [9]*National Cancer Institute, Bethesda, Maryland 20892, USA.*

**Summary** A panel review of histologic specimens was carried out as part of a multi-centre case-control study of gastric cancer (GC) and diet. Comparisons of diagnoses of 100 GCs by six pathologists revealed agreement in histologic classification for about 70–80% of the cancers. Concordance was somewhat higher when using the Lauren rather than the Ming or World Health Organization classification systems. Histologic types from reading biopsy tissue agreed with those derived from surgical specimens for 65–75% of the 100 tumours. Intra-observer agreement in histologic classification, assessed by repeat readings up to 3 years apart by one pathologist, was 95%. The findings indicate that, although overall concordance was good, it is important to standardise diagnoses in multi-centre epidemiologic studies of GC by histologic type.

A large multi-centre case-control study of gastric cancer (GC) was recently conducted in high and low-risk areas in Italy to investigate reasons for the wide regional differences in GC mortality. The study design and protocol have been published elsewhere (Buiatti *et al.*, 1989*a*). Briefly, the case-control study involved seven centres grouped into four areas, two with high (1: Forli/Cremona/Imola and 2: Firenze/Siena) and two with low (3: Genova and 4: Cagliari) death rates for GC. All patients with histologically confirmed GC first diagnosed between June 1985 and December 1987 among residents aged 75 or less in the study areas were eligible as cases and sought for interview.

Pathologists in seven centres provided histologic confirmation for the 1016 newly diagnosed GC case included in the analysis, and classified each case according to histologic type. To evaluate the reproducibility of histologic classifications across centres, a structured panel review of cases was carried out. This paper presents the results of the panel review, assessing inter and intra-observer variability in the histologic classification of GC. This background information is valuable for the evaluation of risk factors according to GC cell types.

### Classification systems

The classification of GC is a complex and difficult task, primarily because different histologic features often coexist in the same tumour. Among the classification systems, the most widely used are those proposed by Lauren, Ming and the World Health Organization (WHO).

Lauren's classification divides GC into two major types: intestinal and diffuse (Lauren, 1965). Intestinal type carcinomas show a definite glandular structure, sometimes with papillae or solid components. The cells lining the glandular lumina resemble those of intestinal neoplasms. The diffuse type is composed of separated single cells or small clusters of cells which diffusely infiltrate the layers of the gastric wall. While the intestinal type is defined on cyto-architectural grounds, the diffuse type is defined by the pattern of growth into the gastric wall. Some carcinomas may not fit into one type or another, and thus fall into 'mixed' or 'unclassified' categories.

Ming (1977) proposed a simple classification of advanced gastric cancer (AGC) based on the growth pattern as an indicator of biological behaviour. AGCs show invasion into or beyond the muscularis propria, and are divided into two types: expanding and infiltrative. The expanding type grows predominantly by expansion, resulting in the production of masses or nodules compressing the surrounding tissues. The infiltrative type is characterised by a diffuse infiltration by individual cells or small groups of cells, without tendency to form masses.

The Lauren and Ming classifications show some overlapping features. In particular, expanding carcinomas are often classified as intestinal type, while infiltrative carcinomas are usually diffuse.

The WHO classification is based on morphological features (Oota & Sobin, 1977). GCs are divided into five categories: adenocarcinoma, adenosquamous carcinoma, squamous cell carcinoma, undifferentiated carcinoma, and unclassified carcinoma. Adenocarcinomas are subdivided into papillary, tubular, mucinous and signet-ring cell types. When different histologic feature are identified in the same specimen, classification is usually based on the predominant pattern, although more than a single cell type can be used to classify more complex cases.

### Materials and methods

### *Selection of histologic material*

Among the GC cases from Florence, included in the multi-centre case-control study, a random sample of 100 GC patients with both endoscopic and surgical specimens were identified. All of the routinely prepared slides available for each subject were retrieved and re-labelled in preparation for the panel review. No information about the age and sex of the patients was provided.

### *Histologic review*

Six pathologists from the centres involved into the case-control study participated in the review of the surgical and biopsy slides. The panel members were from the pathology

Correspondence: E. Buiatti, Epidemiology Unit, CSPO, Viale Volta 171, 50131 Firenze, Italy.

Received 28 September 1990; and in revised form 4 December 1990.

departments in Cagliari (two pathologists), Florence, Forlì, Imola and Siena. Two preliminary discussions were held to define the protocol of the panel review and the histologic classifications to be used. A small group of slides showing typical morphologic patterns according to the three classifications was reviewed. No material was exchanged among centres. For 2 days panel members met to independently classify the specimens.

The pathologists were asked to define each of the 100 surgical specimens as early gastric cancer (EGC) or AGC and to classify them according to the Lauren, Ming and WHO classifications (using only AGCs for the Ming classification). For the Ming classification, a 'mixed' category was introduced to allow classification of specimens in which the two patterns of growth were equally represented. For the WHO classification, panel members were allowed to report more than a single type. The pathologists then independently examined the 100 biopsy specimens, using only the Lauren system to classify histology.

*Analysis*

The diagnoses of each pathologist were compared to those of each of the other members of the panel. No attempt was made to define a 'true' diagnosis or a consensus for an individual specimen. As a measure of agreement between the 15 possible pairs of observers, the kappa statistic was used (Fleiss, 1981). The kappa does not require any assumption concerning the correct diagnosis and includes a correction for the amount of agreement which would be expected by chance alone. Values of kappa near zero indicate chance agreement only, while values near the maximum of 1 indicate perfect agreement. Using the surgical specimens, comparability was measured across three Lauren categories (intestinal, diffuse, and mixed + unclassified), three Ming categories (expanding, infiltrative and mixed), and four combined WHO categories (tubular, papillary and mucinous adenocarcinomas; signet-ring adenocarcinoma; undifferentiated carcinoma; and other types). Considering the diagnosis based on surgical specimens as the 'true' diagnosis or standard, the sensitivity and specificity of each pathologist's diagnoses of the biopsy specimens were estimated for the main Lauren histologic types.

A measure of intra-observer variability, which was not provided by the panel review (no material was recirculated during the meeting), was estimated for one member of the panel (S.B.), who originally diagnosed all specimens from Florence. At the end of the study S.B. independently reclassified these specimens and those from other centres using the Lauren system.

### Results

The distribution of histological diagnoses of the surgical specimens according to the Lauren, Ming and WHO classification systems is shown for each pathologist in Table I. Using the Lauren system, the relative frequencies of intestinal type cancers ranged from 54–72%, while those of the diffuse type varied from 10–31%. The pathologists judged 80–88% of the tumours to be AGC. Among these, from 44–65% were called expanding type and 25–47% were infiltrative using the Ming classification. The dominant histologic type in the WHO system was adenocarcinoma (tubular/papillary/mucinous), accounting for 55–74% of the diagnoses.

Concordance of diagnoses for all 15 possible pairs of pathologists was assessed for each classification system. Exact agreement across the Lauren categories ranged from 68–83%, with kappa values from 0.38–0.70 (median 0.48). Disagreements between pairs of pathologists primarily involved specimens classified by one pathologist as mixed or unclassified and by the other as one of the two major types (intestinal or diffuse); only rarely did one of the pathologists classify a particular specimen as intestinal type while the other classified it as diffuse. Table II shows one example of the concordance in diagnoses, the comparison of Lauren histologies for pathologists A and C.

**Table I** Distribution of 100 surgical specimens of gastric cancer by cell type, by each of six panel pathologists, under the Lauren, Ming and WHO classification systems[a]

| *Classification* | *Pathologists* | | | | | |
|---|---|---|---|---|---|---|
| *Lauren* | *A* | *B* | *C* | *D* | *E* | *F* |
| Intestinal | 60 | 54 | 66 | 62 | 72 | 71 |
| Diffuse | 29 | 31 | 22 | 14 | 10 | 13 |
| Mixed | 10 | 14 | 4 | 23 | 18 | 11 |
| Unclassified | 1 | 1 | 8 | 1 | – | 5 |
| Total | 100 | 100 | 100 | 100 | 100 | 100 |
| *Ming* | *A* | *B* | *C* | *D* | *E* | *F* |
| Expanding | 47 | 36 | 38 | – | 40 | 53 |
| Infiltrative | 41 | 36 | 34 | – | 21 | 26 |
| Mixed | – | 10 | 8 | – | 22 | 2 |
| Total (AGC) | 88 | 82 | 80 | 84 | 83 | 81 |
| *WHO* | *A* | *B* | *C* | *D* | *E* | *F* |
| Adenocarcinoma[b] | 72 | 55 | 64 | 62 | 70 | 74 |
| Signet Ring | 9 | 12 | 9 | 3 | 2 | 19 |
| Undifferentiated | 16 | 19 | 21 | 16 | 13 | 5 |
| Other[c] | 3 | 14 | 6 | 19 | 15 | 2 |
| Total | 100 | 100 | 100 | 100 | 100 | 100 |

[a]Ming's classification only for AGC, and not available for reader D. [b]Tubular, papillary and mucinous adenocarcinoma. [c]All other types.

**Table II** Concordance in Lauren diagnoses of gastric cancer between pathologists A and C, based on 100 surgical specimens

| | *Pathologist C* | | | |
|---|---|---|---|---|
| *Pathologist A* | *Intestinal type* | *Diffuse type* | *Mixed + Unclassified* | *Total* |
| I | 56 | 4 | 6 | 66 |
| D | 2 | 18 | 2 | 22 |
| M | 2 | 7 | 3 | 12 |
| Total | 60 | 29 | 11 | 100 |

Percentage agreement: 77%. Kappa: 0.56.

For the Ming system, agreement between pairs of pathologists was not quite as high, ranging from 57–73%, with kappas from 0.31–0.55 (median 0.41). For the WHO system (with four combined categories), agreement ranged from 68–79% with kappas from 0.34–0.64 (median 0.51). Kappa values (for all three systems) were significantly ($P<0.01$) different from zero. Exact agreement between pairs of pathologists in diagnosing EGC *vs* AGC ranged from 87–96%, with kappas from 0.51–0.86 (median 0.76).

Comparisons of the Lauren diagnoses in the surgical *vs* the biopsy specimens revealed exact agreement ranging from 65% for one pathologist to 75% for another, with kappas ranging from 0.33–0.51. There were no consistent patterns in the types of disagreement, with the percentages of intestinal, diffuse and mixed types about the same whether from a surgical specimen or biopsy. The sensitivities of the diagnoses based on endoscopy ranged from 77–85% for detecting intestinal types, 29–69% for diffuse types, and 0–56% for mixed types. The rate of false positives of the biopsy diagnoses ranged from 13–31% for the intestinal type, 30–50% for the diffuse type, and 59–100% for the mixed type. The rate of false negatives of the biopsy diagnoses ranged from 24–45% for the intestinal type, 5–16% for the diffuse type, and 8–17% for mixed type. Table III shows these levels of agreement for reader C.

Intra-observer variability was assessed for one pathologist (S.B.). Among the 805 GC cases reviewed, 55% were classified as intestinal, 23% as diffuse, 7% as mixed, and 15% as unclassified according to Lauren. For 370 of the GC specimens, S.B. made the original diagnosis prior to the panel review. Concordance between the two repeat readings was extremely high (95%) for these specimens, with a kappa of 0.91 (Table IV). In contrast, for the remaining 435 GC

**Table III** Comparison between Lauren's classification based on endoscopic biopsy and surgical specimens in 100 cases of gastric cancer; pathologist C

| | Surgical specimen | | | |
|---|---|---|---|---|
| *Endoscopic biopsy* | *Intestinal* | *Diffuse* | *Mixed + Unclassified* | *Total* |
| Intestinal | 56 | 4 | 4 | 64 |
| Diffuse | 4 | 14 | 3 | 21 |
| Mixed + Unclassified | 6 | 4 | 5 | 15 |
| Total | 66 | 22 | 12 | 100 |
| Sensitivity | 84.9% | 63.6% | 41.7% | |
| Specificity | 76.5% | 91.0% | 88.6% | |

Percentage agreement: 75%. Kappa: 0.51.

**Table IV** Agreement in Lauren diagnoses of gastric cancer between original classification by pathologist and final reclassification by one study pathologist (S.B.)

(A) Florence

| *Original* | | *Reclassification* | | |
|---|---|---|---|---|
| *classification* | *Intestinal* | *Diffuse* | *Mixed + unclassified* | *Total* |
| Intestinal | 218 | 1 | 4 | 223 |
| Diffuse | 2 | 84 | 4 | 90 |
| Mixed + unclassified | 5 | 3 | 49 | 57 |
| Total | 225 | 88 | 57 | 370 |

Percentage agreement: 95%. Kappa: 0.91.

(B) Other centres

| *Original* | | *Reclassification* | | |
|---|---|---|---|---|
| *classification* | *Intestinal* | *Diffuse* | *Mixed + unclassified* | *Total* |
| Intestinal | 218 | 20 | 74 | 312 |
| Diffuse | 5 | 50 | 21 | 76 |
| Mixed + unclassified | 8 | 20 | 19 | 47 |
| Total | 231 | 90 | 114 | 435 |

Percentage agreement: 66%. Kappa: 0.39.

specimens, 66% (kappa 0.39) of the diagnoses by S.B. agreed with those of the original diagnoses made at the other centres.

### Discussion

Reproducibility of histologic classification in a multi-centre epidemiologic study is of special concern when major hypotheses are linked to particular histologic types, as is the case for GC. Several studied suggest that risk factors for GC may vary by cell type, with the intestinal type showing substantial geographic and demographic variation and suspected to be more closely linked to environmental exposures (Nomura, 1982). The present investigation shows that whereas intra-observer repeatability was excellent, there was a lower rate of agreement between pathologists in specifying histologic types by the widely used Lauren classification, with diagnoses between pairs of pathologists being concordant about 70–80% of the time. Utilising either the Ming or WHO classification systems did not reduce the inter-observer variability.

Our panel review also showed that histologic diagnoses based on surgical *vs* biopsy specimens from the same subjects also agreed about 65–75% of the time. Hence, variations in the availability of surgical compared to biopsy material across centres could also influence the classification of GC. No systematic differences between diagnoses based on surgery *vs* endoscopy were evident, however, so using diagnoses based on either procedure should not differentially bias results. A previous review of 297 GC patients in Florence reached a similar conclusion (Amorosi *et al.*, 1988).

As a preliminary step to using Ming's classification, all surgical specimens were classified as EGC or AGC. Although the pathologists generally tended to agree, the distinctions between early and advanced cancers were not always clear, and levels of concordance were somewhat lower than expected.

As a result of inter-observer discrepancies, it was decided that the final reclassification of all histologic material available in the multi-centre study should be made by a single pathologist. This approach was felt to be necessary to avoid variability between centres which could distort or obscure the detection of GC risks by histologic type.

This study was supported by: Consiglio Nazionale delle Ricerche, Applied Project 'Oncologia', contracts 87.01506.44/87.01581.44/87.1344.44; US National Cancer Institute, contract NO1.CP.51019; Istituto Oncologico Romagnolo, Forli, Italy; Regione Toscana, Italy; Regione Emilia Romagna, Italy; Lega Italiana per la Lotta contro i Tumori, Roma, Italy.

### References

AMOROSI, A., BIANCHI, S., BUIATTI, E., CIPRIANI, F., PALLI, D. & ZAMPI, G. (1988). Gastric cancer in a high-risk area in Italy: Histopathologic patterns according to Lauren's classification. *Cancer*, **62**, 2191.

BUIATTI, E., PALLI, D., DECARLI, A. & 11 others (1989). A case-control study of gastric cancer and diet in Italy. *Int. J. Cancer*, **44**, 611.

FLEISS, J.L. (1981). *Statistical Methods for Rates and Proportions*. 2nd ed. John Wiley: New York.

LAUREN, P. (1965). The two histological main types of gastric carcinoma: diffuse and so-called intestinal-type carcinoma. *Acta Pathol. Microbiol. Scand.*, **64**, 31.

MING, S. (1977). Gastric carcinoma: A pathobiological classification. *Cancer*, **39**, 2475.

NOMURA, A. (1982). Stomach. In Schottenfeld, D. & Fraumeni, J. Jr. (eds), *Cancer Epidemiology and Prevention*, p. 624. W.B. Saunders: Philadelphia.

OOTA, K. & SOBIN, L.H. (1977). Histological typing of gastric and oesophageal tumors. *International Histological Classification of Tumours*. n. 18. WHO: Geneva.